# Status of B-Vitamins and Homocysteine in Diabetic Retinopathy: Association with Vitamin-B12 Deficiency and Hyperhomocysteinemia

**DOI:** 10.1371/journal.pone.0026747

**Published:** 2011-11-01

**Authors:** Alleboena Satyanarayana, Nagalla Balakrishna, Sujatha Pitla, Paduru Yadagiri Reddy, Sivaprasad Mudili, Pratti Lopamudra, Palla Suryanarayana, Kalluru Viswanath, Radha Ayyagari, Geereddy Bhanuprakash Reddy

**Affiliations:** 1 Biochemistry, National Institute of Nutrition, Hyderabad, India; 2 Pushpagiri Vitreo Retina Institute, Hyderabad, India; 3 Ophthalmology, University of California, San Diego, San Diego, California, United States of America; Universidad Peruana Cayetano Heredia, Peru

## Abstract

Diabetic retinopathy (DR) is a common cause of blindness. Although many studies have indicated an association between homocysteine and DR, the results so far have been equivocal. Amongst the many determinants of homocysteine, B-vitamin status was shown to be a major confounding factor, yet very little is known about its relationship to DR. In the present study, we, therefore, investigated the status of B-vitamins and homocysteine in DR. A cross-sectional case–control study was conducted with 100 normal control (CN) subjects and 300 subjects with type-2 diabetes (T2D). Of the 300 subjects with T2D, 200 had retinopathy (DR) and 100 did not (DNR). After a complete ophthalmic examination including fundus fluorescein angiography, the clinical profile and the blood levels of all B-vitamins and homocysteine were analyzed. While mean plasma homocysteine levels were found to be higher in T2D patients compared with CN subjects, homocysteine levels were particularly high in the DR group. There were no group differences in the blood levels of vitamins B1 and B2. Although the plasma vitamin-B6 and folic acid levels were significantly lower in the DNR and DR groups compared with the CN group, there were no significant differences between the diabetes groups. Interestingly, plasma vitamin-B12 levels were found to be significantly lower in the diabetes groups compared with the CN group; further, the levels were significantly lower in the DR group compared with the DNR group. Higher homocysteine levels were significantly associated with lower vitamin-B12 and folic acid but not with other B-vitamins. Additionally, hyperhomocysteinemia and vitamin-B12 deficiency did not seem to be related to subjects' age, body mass index, or duration of diabetes. These results thus suggest a possible association between vitamin-B12 deficiency and hyperhomocysteinemia in DR. Further, the data indicate that vitamin-B12 deficiency could be an independent risk factor for DR.

## Introduction

Diabetic retinopathy (DR) is one of the most common microvascular complications of diabetes and ranks as a common cause of blindness worldwide [1], [2]. Diabetic retinopathy could become a major threat to public health in the future due to the global prevalence of diabetes, which is projected to affect 438 million people by 2030 [3]. Both the duration of diabetes and its metabolic control have been identified as the risk factors most strongly associated with the development of DR [4], [5]. Diabetic retinopathy occurs in 70% of all persons having diabetes for more than 15 years. While the prevalence of DR has varied (20%–60%) in different studies, a recent study indicated that the estimated prevalence was 28.5% among US adults [6]. The prevalence of DR among urban subjects with diabetes in India was reported to be about 17% [7], whereas in a clinical study it was found to be 34% among patients with type 2 diabetes (T2D) [8]. The prevalence of DR in South India was reported to be 22.4% based on the Andhra Pradesh Eye Disease Study (APEDS) of self-reported diabetes [9]. The prevalence of DR was 0.5% in the general rural populations of South India and 10.5% among patients with diabetes [10].

Diabetic retinopathy is characterized by the appearance of vascular lesions of increasing severity, culminating in the growth of new vessels. Early or nonproliferative DR (NPDR) is marked by retinal vascular microaneurysms, blot hemorrhages, cotton-wool spots, loss of retinal pericytes, increased vascular retinal permeability, alterations in regional blood flow, and abnormal retinal microvasculature, all of which lead to retinal ischemia. Proliferative DR (PDR), the more severe state, is marked by the formation of abnormal, fragile new blood vessels that are prone to hemorrhage [11], [12].

Although the prevalence of DR increases with the duration of diabetes, studies have shown that intensive glycemic control can delay its development [4], [5]. In principle, all patients with diabetes might be expected to develop diabetic microvascular complications if hyperglycemia alone were the triggering factor for diabetic complications. It is, however, noteworthy that some patients may still develop DR even with good glycemic control. Conversely, some patients with poor glycemic control avoid this complication, notably long-surviving patients with type-1 diabetes (T1D). Therefore, the impact of strict glycemic control on prevention of diabetic complications is not that scrupulous [4], [5], [13]. Multiple factors are likely to be involved in predisposing diabetes subjects to complications, as evidenced by many but not all patients with diabetes developing one or more microvascular complications. If the predisposing factors are known, it may be possible to delay the onset and progression of these complications. Hence, there is an obvious need to understand the risk factors that are associated with diabetic complications.

Although genetic susceptibility appears to be the primary predisposing factor for DR (reviewed by Ng, 2010 [14] and Fu et al, 2010 [15]; [16]), the role of environmental factors like nutritional and dietary factors are not to be discounted. Vitamins and mineral supplementation for the management of T2D has been reported [17], but its role in the prevention and development of T2D in general and diabetic complications in particular has not been established clearly. Further, diabetes itself can alter the nutritional status [18], [19], and experiments suggest that patients with diabetes are prone to deficiency of micronutrients such as magnesium, zinc, copper, manganese, and chromium. It was observed that serum ascorbic acid, B-vitamins, and possibly 1,25-dihydroxycholcalciferol concentrations are low in diabetic patients. Studies indicate low plasma thiamine (vitamin B1) in both T1D and T2D patients [20], and high-dose thiamine and its derivatives such as benfotiamine can prevent the development of microvascular complications [21]. However, the levels of vitamin B1 in DR have not been investigated so far.

Low concentrations of folic acid and other B vitamins are associated with increased risk of vascular damage through homocysteine. Homocysteine has been extensively studied in recent years as a biomarker as well as a risk factor for vascular diseases, including vaso-occlusive diseases of the eye [22]–[26]. Homocysteine is a by-product of transmethylation reactions and detoxified by methionine synthetase, which is dependent on vitamin B12 and folate as coenzymes for its proper function [27], [28]. Determinants of hyperhomocysteinemia such as low concentrations of folate and B-vitamin coenzymes and altered activities of enzymes involved in the breakdown of homocysteine are also associated with increased risk of cardiovascular complications [26]. Nevertheless, B-vitamin status and its contribution to hyperhomocysteinemia in DR have not been examined. Therefore, we investigated the status of B-vitamins (B1, B2, B6, B12, folic acid) and homocysteine in DR.

## Results

This is a hospital-based case-control study consisting of T2D subjects with (DR) and without retinopathy (DNR) along with normal control (CN) subjects. The characteristics of the CN, DNR, and DR groups are shown in Table 1. The sex distribution was approximately the same in all the groups (male and female were, respectively, 58% and 42% in CN, 56% and 44% in DNR, and 55% and 45% in DR). Further, there was no significant difference between male and female subjects in all three groups with respect to demographics and measured parameters. Therefore, the data for both men and women were pooled as a whole in the respective groups. Mean age, body mass index (BMI), and hemoglobin levels were comparable between the groups. The duration of diabetes was matched for DNR and DR (p>0.05). Amongst diabetes groups, glycosylated hemoglobin (HbA1c) levels were higher in DR compared to DNR (p<0.05). While plasma total cholesterol and low-density lipoprotein (LDL) were comparable between the groups, triglyceride levels were higher and high-density lipoprotein (HDL) was lower in the diabetes groups compared with the CN group (Table 1). Nevertheless, the levels of triglycerides and HDL were comparable between the DNR and DR groups.

**Table 1:** Clinical and demographic profile of control (CN) and diabetes patients without (DNR) and with retinopathy (DR).

Parameter	Normal(n = 100)	DNR(n = 100)	DR(n = 194)	F –Value	P –Value
**Age** (years)	53.99±9.22^a^	54.76±9.29^a^	55.75±8.24^a^	2.1	0.128
**BMI**	23.63±3.04^a^	25.21±4.19^a^	24.32±4.45^a^	1.6	0.212
**Hb** (g/dL)	14.89±1.73^a^	14.31±2.16^a^	14.11±2.32^a^	1.4	0.255
**Duration** (years)	-	10.16±6.92^a^	11.03±6.92^a^	2.2	0.098
**Glucose** (mg/dL)	99.16±18.86^a^	209.97±85.10^b^	221.28±91.36^b^	146.8	0.000
**HbA1c** (%)	5.64±1.14^a^	8.94±2.49^b^	10.33±2.94^c^	86.0	0.000
**Insulin** (µU/mL)	29.14±9.94^a^	38.05±22.71^a^	34.75±19.58^a^	1.4	0.255
**Total Cholesterol** (mg/dL)	169.39±39.65^a^	167.03±53.56^a^	178.01±58.47^a^	0.6	0.552
**Triglycerides** (mg/dL)	129.78±63.57^a^	151.99±65.43^b^	163.00±77.90^b^	4.3	0.015
**HDL** (mg/dL)	35.02±8.81^a^	30.09±9.21^b^	28.70±7.41^b^	8.8	0.000
**LDL** (mg/dL)	112.69±30.13^a^	110.66±29.01^a^	117.11±34.05^a^	0.6	0.570

Note: 1. Values are Mean ± SD.

2. Variables of glucose, HbA1c and TC were transformed into logarithmic values due to heterogeneity of variances across groups and mean values across groups were compared by oneway ANOVA ‘F’ test with post hoc test of Tukey's multiple comparisons.

3. Significant differences (p<0.05) of mean values between the groups are indicated by different superscript letters.

**Figure 1:**
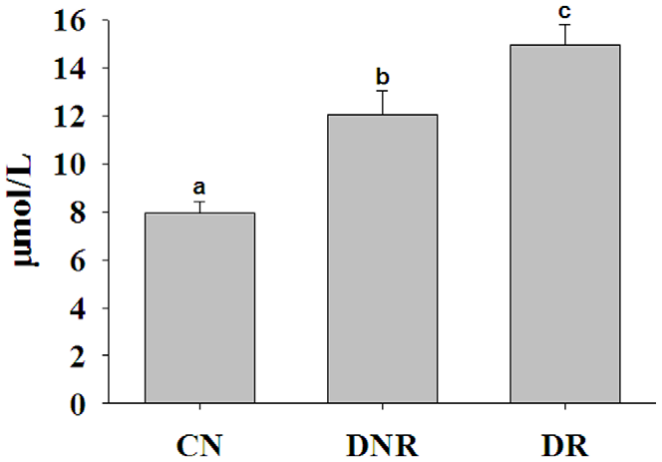
Plasma homocysteine levels. Data represent mean ± SE in control (CN; n = 75) and diabetes patients without (DNR; n = 75) and with retinopathy (DR; n = 150). Data were transformed into log values and compared mean values across groups by oneway ANOVA ‘F’ test with post hoc test of Tukey's multiple comparisons. Significant differences (p<0.05) of mean values between the groups are indicated by different letters on the bars after adjusting the duration of diabetes between DNR and DR.

**Figure 2:**
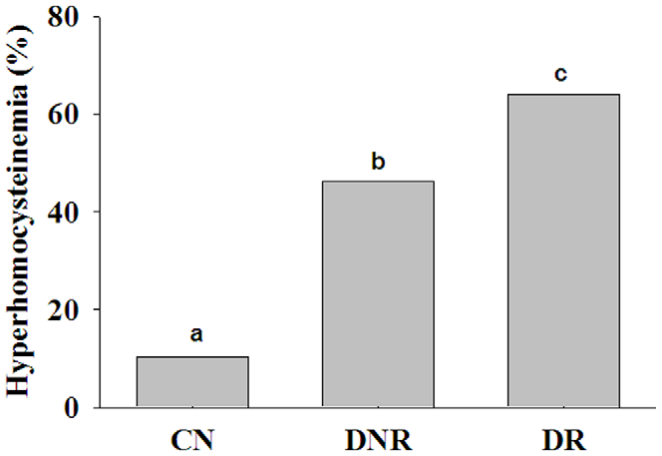
Prevalence (%) of hyperhomocysteinemia (>12 µmol/L) in control (CN) and diabetes patients without (DNR) and with retinopathy (DR). Data indicate percent of subjects above 12 µmol/L of the respective group. Significant differences (p<0.05) of mean values between the groups are indicated by different letters on the bars after adjusting the duration of diabetes between DNR and DR.

The mean plasma homocysteine levels were significantly higher in the DNR group compared with the CN group, and a further increase (p<0.05) was found in the DR group compared with the DNR group (Figure 1). However, there was a considerable overlap of hyperhomocysteinemia between the DNR and DR groups. Hence, we determined the prevalence of hyperhomocysteinemia (>12 µmol/L), which was significantly different (p<0.01) between the groups: 65% in DR, 46% in DNR, and 11% in CN (Figure 2). Further, we also compared the homocysteine levels in DR patients with a shorter duration of diabetes (<5 years) with those of DNR patients with a long duration of diabetes (>5 years). The mean value of homocysteinemia in DNR of >5 years duration was 12.4 µmol/L, while it was 14.2 µmol/L in DR of <5 years duration. In addition, when duration was controlled for in both DNR and DR groups, DR patients had significantly higher levels of homocysteine than DNR patients. Together, these results suggest an association between hyperhomocysteinemia and DR.

**Figure 3:**
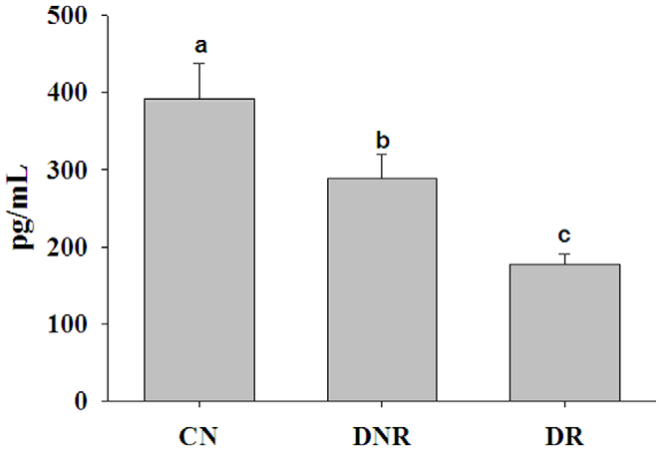
Plasma vitamin-B12 levels. Data represent mean ± SE in control (CN; n = 75) and diabetes patients without (DNR; n = 75) and with retinopathy (DR; n = 150). Data were transformed into log values and compared the mean values across groups by oneway ANOVA ‘F’ test with post hoc test of Tukey's multiple comparisons. Significant differences (p<0.05) of mean values between the groups are indicated by different letters on the bars.

**Figure 4:**
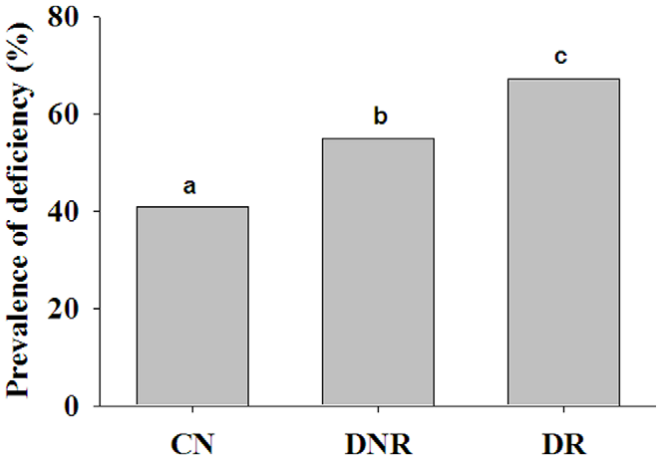
Prevalence (%) of vitamin-B12 deficiency in control (CN) and diabetes patients without (DNR) and with retinopathy (DR). Data indicate percent of subjects below 200 pg/mL of the respective group. Proportion Z test was done to compare prevalence between groups. Significant differences (p<0.001) of mean values between the groups are indicated by different letters on the bars.

Since B-vitamins are linked to homocysteine metabolism and homeostasis, we then determined the B-vitamin levels. The vitamin B1 levels were marginally (but statistically significantly) higher in DNR compared with the CN group, but there was no significant difference between the DNR and DR groups. Furthermore, vitamin B1 levels were comparable between the DR and CN groups. While the blood levels of vitamin B2 were comparable between the groups, plasma vitamin B6 levels were significantly lower (p<0.05) in the diabetes groups (DNR and DR) compared with the CN group (Table 2). In spite of the deficiency of vitamin B6 in the diabetes groups, there was no significant difference in the mean levels of vitamin B6 between the DNR and DR groups (Table 2). Similarly, plasma folic acid levels were lower but not deficient in the diabetes groups (DNR and DR) compared with the CN group; however, the levels were comparable between the DNR and DR groups (Table 2). Plasma vitamin B12 levels were significantly lower (p<0.01) in diabetes patients (DNR and DR) compared with the CN subjects (Figure 3). Interestingly, in this study significantly lower (p<0.05) plasma vitamin B12 levels were observed in DR patients compared with DNR patients (Figure 3). The mean vitamin B12 levels in the DR group were below the normal range (200–1000 pg/mL). However, the vitamin B12 levels ranged from 20 to 1500 pg/mL with considerable overlap between the groups. Therefore, we examined the data for prevalence of vitamin B12 deficiency in the three groups. With 200 pg/mL as the cut-off level for deficiency [29]–[31], the prevalence of vitamin B12 deficiency was significantly different (p<0.001) between the groups; 67% in DR, 54% in DNR, and 41% in CN (Figure 4). The ratio of vitamin B12 to folic acid was also significantly different (p<0.05) between CN and DR groups, but not between the DNR and DR groups (data not shown). However, there was no significant difference in homocysteine and vitamin B12 levels between NPDR and PDR patients (14.72 vs. 14.11 µmol/L and 182 vs. 177 pg/mL, respectively). These results suggest that DR is associated with a higher prevalence of vitamin B12 deficiency.

**Table 2:** Blood and plasma levels of vitamins B1, B2, B6 and folic acid of control (CN) and diabetes patients without (DNR) and with retinopathy (DR).

Vitamin	CN	DNR	DR	F Value	P-values
**B1** **(ng/mL)**	67.6±3.1^a^n = 45	70.1±3.8^b^n = 45	67.2±3.0^ab^n = 45	3.9	0.024
**B2** **(ng/mL)**	231±9.3^a^n = 45	248±8.2^a^n = 45	238±7.0^a^n = 45	1.99	0.143
**B6** **(ng/mL)**	20.6±1.3^a^n = 45	13.0±1.1^b^n = 45	14.6±1.0^b^n = 60	10.1	0.000
**Folic acid** **(ng/mL)**	10.0±0.9^a^n = 75	7.8±0.6^ab^n = 75	7.2±0.4^b^n = 150	4.7	0.009

Note: 1. Values are Mean ± SD.

2. Mean values across groups were compared by oneway ANOVA ‘F’ test with post hoc test of Tukey's multiple comparisons.

3. Significant differences (p<0.05) of mean values between the groups are indicated by different superscript letters.

Homocysteine levels were inversely related (p<0.05) to vitamin B12 and folic acid but not to vitamins B1, B2, and B6, when data of all the groups were considered (Table 3). Interestingly, irrespective of groups, homocysteine levels were significantly (p<0.05) associated with glucose and HbA1c levels but not related to subjects' age, BMI, or duration of diabetes (Table 3). Likewise, vitamin B12 levels were significantly (p<0.05) but inversely associated with glucose, HbA1c, and homocysteine and positively related with folic acid but not with age, BMI, or duration (Table 3). Considering that plasma folic acid levels were low but not deficient and glucose levels in diabetes groups were comparable irrespective of the presence of DR, the odds ratio for vitamin B12 is twice (95% CI, 1.1–3.7) in the DR group compared with the DNR group after adjusting for age and duration of diabetes.

**Table 3:** Correlations of vitamin-B12 and homocysteine with demographic and other biochemical parameters.

Parameter	Vitamin-B12		Homocysteine	
	r-value	p-value	r-value	p-value
**Age**	0.111	0.055	0.088	0.328
**Duration**	0.074	0.263	0.162	0.113
**BMI**	0.082	0.292	0.083	0.493
**Glucose**	−0.177	0.016	0.444	0.000
**HbA1c**	−0.192	0.034	0.247	0.047
**Vitamin -B1**	−0.064	0.571	0.294	0.066
**Vitamin -B2**	−0.062	0.577	0.284	0.056
**Vitamin -B6**	0.047	0.614	−0.137	0.383
**Folic acid**	0.473	0.000	−0.323	0.002
**Vitamin -B12**	-	-	−0.485	0.000
**Homocysteine**	−0.485	0.000	-	-

Correlations (r-value) were assessed by Spearman rank correlation. While positive r-value indicates direct correlation, negative r-value indicates inverse relationship between the variables.

## Discussion

Approximately 5% of the global prevalence of blindness is considered to be due to DR, with estimates of 15%–17% in developed countries [1]. Nutritional status, particularly micronutrients, may affect the risk of DR by influencing the biochemical mechanisms underlying DR. A biochemical indicator that has generated considerable interest as a risk factor for many vascular diseases, including DR, is homocysteine. Although many studies have evaluated the association between homocysteine and DR, the results are varied and inconsistent [22], [25], [32]–[34]. Amongst the many determinants of homocysteine, B-vitamin status is a major confounding factor [23], [28], [35], [36]. However, very little is known about the extent to which B-vitamin status is associated with DR vis-à-vis homocysteine. Considering the general prevalence of micronutrient deficiency and its contribution to many metabolic disorders, such as intrauterine growth retardation, diabetes, and cardiovascular diseases in India [29]–[31], determining the status of micronutrients with regard to the prevalence and pathogenesis of DR is critical. Therefore, we evaluated the association of B-vitamin status and homocysteine with DR in a systematic way. To the best of our knowledge, a relationship between B-vitamin status, homocysteine, and DR has so far not been reported.

The results of the present study show that while lower levels of folic acid and vitamins B6 and B12 were observed in patients with diabetes irrespective of the presence of retinopathy, only vitamin B12 deficiency was associated with DR. Interestingly, a significant association was revealed between DR and hyperhomocysteinemia based on plasma homocysteine levels. Hyperhomocysteinemia has several causes, including dietary deficiencies of folic acid and vitamins B6 and B12. Furthermore, supplementation of folic acid and vitamin B12 is known to reduce homocysteine levels [23], [26]. Lack of vitamin B12 is thought to be a more important determinant for increased homocysteine, particularly in older people, and it becomes the limiting nutrient for maintaining normal plasma concentrations once folate levels are optimized. It should be noted in the present study that the mean age of the subjects was 55 years and the folate levels were still in the normal range, although lower mean levels were found in patients with diabetes. In addition, plasma homocysteine in diabetes varies depending on the presence or absence of nephropathy. However, in this study the increased levels of homocysteine were independent of renal failure because we excluded DNR and DR patients with renal complications. Therefore, lower vitamin B12 status appears to be a determining factor for increased homocysteine in DR patients in this population. Further, vitamin B12 levels did not seem to be influenced by patients' age, BMI, or duration of diabetes because the associations between vitamin B12 and these variables were not statistically significant. Moreover, vitamin B12 deficiency is two times as likely in the DR group compared with the DNR group after adjusting for age and duration of diabetes. Together, these results also suggest that vitamin B12 deficiency could be an independent risk factor for DR.

The Age-Related Eye Disease Study (AREDS) has shown that antioxidant and trace element micronutrients can reduce the risk of developing age-related macular degeneration [37]–[39]. Supplementation with some antioxidants and micronutrients, including vitamin B1 (benfotiamine), has shown encouraging results in experimental models of DR and human studies, though the findings of clinical trials with these antioxidant micronutrients have been less conclusive [40]–[42]. Interestingly, a study showed that AREDS-based micronutrients proven to be beneficial in ameliorating the lesions associated with DR in experimental rats [43]. Although we have yet to analyze the association of micronutrients other than B-vitamins with DR, the results reported here imply that a deficiency or inadequacy of vitamin B12 may predispose diabetes patients to DR. Lowered levels of cobalt, an integral component of vitamin B12 (cyanocobalamine), paired with decreased dietary intake of vitamin B12 in the DR group compared with the DNR group further supports the above implication (GBR unpublished data). It should be noted that low levels of vitamin B12 have been recognized in Indians for a long time and recent studies confirm low concentrations of vitamin B12 and the implications for diabetes and cardiovascular diseases in India [29], [30], [44]. Our study also further substantiates the general prevalence of vitamin B12 deficiency in India; about 40% adults above 50 years are deficient and the prevalence is much higher in patients with diabetes. This is the first study to show an association of B-vitamins with DR, and more controlled prospective studies are warranted to confirm the role of vitamin B12 deficiency in the development of DR.

## Methods

### Study design, subjects, and sample collection

This is a hospital-based case–control study conducted during April 2008 to March 2010 consisting of 300 T2D patients either with or without retinopathy (DR, n = 200 and DNR, n = 100, respectively). In addition, we recruited 100 control (CN) subjects consisting of partners, relatives, and friends of patients and employees of the National Institute of Nutrition matched for similar socioeconomic status of the T2D patients. The CN group consisted of asymptomatic subjects of age 50 years and above without any history of cardiovascular and renal complications. Subjects with T2D with and without retinopathy were recruited from patients attending the Pushpagiri Vitreo Retina Institute and were matched for duration of diabetes. Control and diabetes subjects on nutritional supplements for the last 6 months and those with a history of nephropathy (based standard renal function tests) and complications other than DR were excluded. However, many subjects with diabetes (DR and DNR) used antidiabetic medication per their physician's advice. History or presence of diabetic complications other than DR was assessed by clinical as well as biochemical methods. All patients with diabetes underwent a complete ophthalmic examination consisting of best corrected visual acuity, slit-lamp biomicroscopy, indirect ophthalmoscopy, and fundus fluorescein angiography. Diabetic retinopathy grading was done using the Early Treatment Diabetic Retinopathy Study adaptation of the modified Airlie House classification system, and DR was further categorized as NPDR and PDR [11], [45], [46]. The study was carried out in accordance with the guidelines of the Helsinki Declaration of 1975 and approved by the Institutional Ethics Committees of Pushpagiri Vitreo Retina Institute and National Institute of Nutrition. After obtaining written informed consent from all participants, venous blood samples were collected in EDTA tubes in the morning following an overnight fast. An aliquot of each whole blood sample was kept, while the remainder was separated into plasma and red blood cells [46].

### Biochemical estimations

Fasting blood glucose was estimated in plasma by the glucose oxidase–peroxidase method using a kit (BioSystems, Barcelona, Spain). Glycosylated hemoglobin (HbA1c) was estimated in whole blood by ion-exchange chromatography using a kit (BioSystems). While plasma insulin was estimated by a radioimmunoassay kit (Board of Radiation and Isotope Technology-Department of Atomic Energy, Mumbai, India), the lipid profile (total cholesterol, triglycerides, HDL) was analyzed using commercially available kits (BioSystems).

### Estimation of homocysteine and B-vitamins

We employed HPLC to estimate vitamins B1, B2, and B6. The levels of vitamin B1 and B2 in whole blood and B6 in plasma were measured as total thiamine pyrophosphate, flavin adenine dinucleotide, and pyridoxal-5′-phosphate, respectively, based on previously reported methods [47] using commercially available HPLC kits (Recipe Chemicals and Instruments GmbH, Germany). Plasma levels of vitamin B12 and folic acid were measured by a solid phase radioimmunoassay method using a commercially available kit designed for simultaneous measurement of these vitamins (Siemens Medical Solutions Diagnostics, Los Angeles, CA, USA). Radioactivity was measured by a gamma counter with a dual channel for determining ^57^Co and ^125^I simultaneously (Perkin Elmer, 3 wizard 1480, USA). Plasma total homocysteine levels were measured by HPLC using a Supelcosil™ LC-18-DB (150 mm by 4.6 mm) column according to methods reported previously [48], [49].

### Statistical analysis

Statistical analysis was performed using SPSS for Windows version 15.0. Mean and SD or SE values of vitamins and homocysteine, BMI, age, and duration of disease were calculated. No outliers were found in the data based on Grubb test. Comparison of mean values of these variables across groups was done by one-way ANOVA *F* test with post hoc Tukey test. Log transformations were also performed to stabilize the normality of the skewed variables for glucose, HbA1c, HDL, homocysteine, and vitamin B12. Proportion *Z* test was used for comparison of prevalence of vitamin B12 deficiency. The relationship between homocysteine and vitamin B12 with age, duration of disease, BMI, glucose, and vitamins B1, B2, B6, and folic acid was calculated by Spearman rank correlation coefficients, and risk was estimated by odds ratio with logistic regression model. Two-tailed test was considered for all statistical tests. The level of significance was p<0.05.

### Sample size for the estimation of vitamins and homocysteine

Although the data for demographic and clinical parameters were collected for all 400 subjects, the actual minimum required sample size for vitamins and homocysteine was determined assuming 95% CI and 80% power and using SD of respective vitamins and homocysteine. Hence the data on vitamins and homocysteine were obtained on a subsample. Nevertheless, the sample size was increased for homocysteine and vitamin B12, for strengthening their association with DR.
